# Epigenetic regulation of Thy-1 gene expression by histone modification is involved in lipopolysaccharide-induced lung fibroblast proliferation

**DOI:** 10.1111/j.1582-4934.2012.01659.x

**Published:** 2013-01-11

**Authors:** Zhengyu He, Xiangrui Wang, Yuxiao Deng, Wen Li, Yongming Chen, Shunpeng Xing, Xianyuan Zhao, Jia Ding, Yuan Gao

**Affiliations:** Department of Anesthesiology, Renji Hospital, Shanghai Jiaotong University School of MedicineShanghai, China

**Keywords:** lung fibroblast proliferation, lipopolysaccharide, thymocyte differentiation antigen 1, histone modification

## Abstract

Lipopolysaccharide (LPS)-induced pulmonary fibrosis is characterized by aberrant proliferation and activation of lung fibroblasts. Epigenetic regulation of thymocyte differentiation antigen 1 (Thy-1) is associated with lung fibroblast phenotype transformation that results in aberrant cell proliferation. However, it is not clear whether the epigenetic regulation of Thy-1 expression is required for LPS-induced lung fibroblast proliferation. To address this issue and better understand the relative underlying mechanisms, we used mouse lung fibroblasts as model to observe the changes of Thy-1 expression and histone deacetylation after LPS challenge. The results showed that cellular DNA synthesis, measured by BrdU incorporation, was impacted less in the early stage (24 hrs) after the challenge of LPS, but significantly increased at 48 or 72 hrs after the challenge of LPS. Meanwhile, Thy-1 expression, which was detected by real-time PCR and Western blot, in lung fibroblasts decreased with increased time after LPS challenge and diminished at 72 hrs. We also found that the acetylation of either histone H3 or H4 decreased in the LPS-challenged lung fibroblasts. ChIP assay revealed that the acetylation of histone H4 (Ace-H4) decreased in the Thy-1 promoter region in response to LPS. In addition, all the above changes could be attenuated by depletion of TLR4 gene. Our studies indicate that epigenetic regulation of Thy-1 gene expression by histone modification is involved in LPS-induced lung fibroblast proliferation.

## Highlights

- Mouse lung fibroblast was challenged with LPS from 0 to 72 hrs.
- DNA synthesis increased at 48 or 72 hrs after LPS challenge.
- Thy-1 expression diminished 72 hrs after LPS challenge.
- Acetylation of either histone H3 or H4 decreased in LPS-challenged lung fibroblasts.
- Epigenetic regulation of Thy-1 is involved in LPS-induced fibroblast proliferation.

## Introduction

The formation and development of pulmonary fibrosis are associated with acute lung injury (ALI) and acute respiratory distress syndrome (ARDS), which is characterized by aberrant proliferation and activation of lung fibroblasts. Lipopolysaccharide (LPS), a component of bacteria membranes, plays an important role in the development of pulmonary fibrosis [1, 2]. Our previous studies revealed that LPS could directly induce lung fibroblast activation and proliferation through Toll-like receptor 4 (TLR4), a specific receptor of LPS and downstream intracellular signal transduction pathways [3, 4]. However, there is considerable controversy regarding the effect of LPS on fibroblast proliferation. Similar studies achieved opposite results from fibroblasts derived from many different tissues or even from different parts of the same tissue [5–10], which suggested that fibroblasts might have heterogeneous subpopulations that play distinct roles in fibrotic responses.

According to the expression status of thymocyte differentiation antigen 1 (Thy-1, CD90) on the cell surface, lung fibroblasts can be divided into two subpopulations: Thy-1-positive [Thy-1 (+)] and Thy-1-negative [Thy-1 (−)] cells. Thy-1, a cell-surface glycoprotein presents on normal lung fibroblasts [11], is absent from the fibroblastic foci of idiopathic pulmonary fibrosis (IPF) [12]. The expression of Thy-1 correlates inversely with fibrogenic phenotypic characteristics. Thereby, it is known as a ‘fibrosis suppressor’. Thy-1 (−) fibroblasts have an increased propensity to fibrogenic responses after various fibrogenic mediator stimulation. Additionally, fibrogenic injury promotes loss of Thy-1 expression in lung fibroblast, which appears to be an important mechanism in lung fibrogenesis [11, 13–15]. It has been reported that epigenetic control, such as histone deacetylation, is involved in the regulation of Thy-1 gene expression [12, 16]. On the other hand, several studies found that LPS could manipulate the epigenetic regulation of gene expression by histone acetylation [17, 18]. However, it is not clear whether the epigenetic regulation of Thy-1 expression is required for LPS-induced lung fibroblast proliferation.

In this study, we observed dynamic changes of Thy-1 expression at the genetic and cellular level in cultured mouse lung fibroblasts from 0 to 72 hrs after being challenged with LPS. Furthermore, we investigated the epigenetic mechanism related to the association of LPS-induced TLR4 and Thy-1-related lung fibroblast phenotype transformation through the depletion of TLR4 gene with RNA interference (RNAi).

## Materials and methods

### Primary cultures of mouse lung fibroblasts

Lung fibroblasts were isolated from a C57/BL6 mouse, as described in our previous study [19]. Briefly, an 8-week-old mouse (Shanghai SLAC Laboratory Animal Co. Ltd., Shanghai, China) was killed by euthanasia. Lung tissues were promptly excised, washed with phosphate buffered saline (PBS) and cut to 1 mm^3^ tissue masses. Tissues were then distributed evenly along the bottom of culture plates and covered with Dulbecco's Modified Eagle's Medium (DMEM) containing 10% calf serum (Gibco, Brooklyn, NY, USA). The plates were cultured at 37°C in a humidified 5% CO2 incubator (Labotect, Gottingen, Germany), and DMEM was changed every 3 days. When the cultures reached 80% confluence, adherent cells were detached by exposure to 0.25% trypsin for 5 min., and then passaged at a 1:4 dilution. Cells grew to a typical fusiform shape after four generations. Fibroblasts were identified by strong positive vimentin immunocytochemical stains (data not shown) as previously described [20], and then passages five through seven were used.

This procedure was approved by the Animal Care and Use Committee of the Shanghai Jiaotong University School of Medicine. All procedures were carried out in accordance with the guidelines for animal care published by the United States' National Institutes of Health (NIH) for animal care (Guide for the Care and Use of Laboratory Animals, Department of Health and Human Services, NIH publication no. 86-23, revised 1985).

### Construction and identification of RNAi lentivirus vector targeting the TLR4 gene

A specific RNAi lentivirus vector targeting TLR4 (TLR4-siRNA-lentivirus) was obtained from Shanghai GeneChem Co. Ltd (Shanghai, China) as our previous study described [19]. The RNA interference target sequences were designed as follows: 5′-aaGTCAATCTCTCTTTAGACA-3′. Double-stranded DNA oligo was synthesized to knocking down mouse TLR4 expression. The oligo was digested with restriction enzymes to yield overhanging sticky ends and inserted into the RNA interference lentivirus vector pGCL-GFP (Shanghai GeneChem Co. Ltd.), and the vector was transformed into competent *Escherichia coli* cells. PCR was used to select positive clones.

The assisted packaging vector plasmids pHelper 1.0 (gag/pol) and pHelper 2.0 (VSVG) encoded lentivirus particles and were prepared with the recombinant virus plasmid pGCL-TLR4siRNA-GFP (Shanghai GeneChem Co. Ltd.). Extractions were outperformed without endotoxin contamination and 293T cells were transfected for virus packing using Lipofectamine2000 (Invitrogen, USA). Lentivirus-TLR4 -siRNA (GENECHEM Co. Ltd., Shanghai, China) was collected after transfection, and virus titres in the contracted 293T cells were detected at 4 × 10^8^ TU/ml.

Primary cultured mouse lung fibroblasts were infected with lentivirus-TLR4-siRNA, or control-siRNA-lentivirus (GFP expression), and were collected 5 days after virus infection. TLR4 expression levels were determined by real-time PCR and Western blots.

### Experimental groups and treatment

**1** Purified mouse lung fibroblasts were seeded into 96-well plates and grown in DMEM containing 10% calf serum in a humidified atmosphere containing 5% CO_2_. When ˜60% confluence was reached, the medium was replaced with serum-free medium and the cultures were incubated for an additional 24 hrs at 37°C in 5% CO_2_. Finally, the serum-free medium was replaced with DMEM containing 10% calf serum and the cells were divided among several groups: TLR4-depleted cells: TLR4-siRNA-lentivirus was added to cells at a concentration of 1 × 10^8^ TU/ml for 48 hrs; and negative controls were established by adding the same amount of negative control-siRNA-lentivirus containing scrambled non-functional RNAi sequences at same time.

**2** LPS challenge: purified LPS (derived from O55:B5 *E. coli*; Sigma-Aldrich, Saint Louis, MO, USA) was added to TLR4-siRNA-lentivirus (or control-siRNA-lentivirus, GENECHEM Co. Ltd.) transfected cells at a concentration of 1 μg/ml and incubated for 72 hrs or at different time-point as indicated in the Figures. Experiments were performed three times in each group.

### Cell proliferation assay

To evaluate the proliferation of lung fibroblasts in response to different treatments, DNA synthesis, a direct indicator of cell proliferation, was measured using BrdU incorporation assay as previously described [21]. Briefly, prior to harvesting, cells were incubated with BrdU for 4 hrs, followed by immunostaining with anti-BrdU antibody, according to the manufacturer's instructions (Cell Signaling Technology, Boston, MA, USA). DNA synthesis was quantified by the magnitude of absorbance (optical density, OD) at 450 nm, which was proportional to the quantity of BrdU incorporated into cells.

### Real-time PCR

To analyse Thy-1 and TLR4 mRNA expression, total RNA was isolated from cells using Trizol reagent (Invitrogen) and the RNeasy kit, followed by reverse transcription using M-MLV polymerase (Promega, Madison, WI, USA). Real-time PCR was performed using sequence-specific primers: GAPDH-F: 5′-TGGTGAAGGTCGGTGTGAAC-3′, GAPDH-R: 5′-GCTCCTGGAAGATGGTGATGG-3′; TLR4-F: 5′-ATGGCATGGCTTACACCACC-3′, TLR4-R: 5′-GAGGCCAATTTTGTCTCCACA-3′; Thy-1-F: 5′-GCCGCCATGAGAATAACA-3′, Thy-1-R: 5′-GCTAGGGTAAGGACCTTGAT-3′. Amplification was performed on the IQ5 PCR System (Bio-Rad, Hercules, CA, USA) with an initial denaturing step at 95°C for 15 sec., 45 cycles of denaturing at 95°C for 5 sec. and annealing at 60°C for 30 sec. Thy-1 and TLR4 gene expression, which was normalized to GAPDH expression, was analysed by the ΔΔCt method [22].

### Western blot analysis

Protein expression of Thy-1, TLR4, total histone H3 (Total-H3), total histone H4 (Total-H4), acetylated H3 (Ace-H3) and acetylated H4 (Ace-H4) was detected by Western blotting. Briefly, cells were collected and lysed with 1× RIPA lysis buffer (50 mM Tris-HCl, pH 7.4, 150 mM NaCl, 1% Nonidet P-40, 0.5% deoxycholic acid, 0.1% sodium dodecyl sulphate (SDS), 5 mM EDTA, 2 mM phenylmethylsulfonyl fluoride (PMSF), 20 μg/ml aprotinin, 20 μg/ml leupeptin, 10 μg/ml pepstanin A, 150 mM benzamidine) on ice for 10–15 min. Supernatants were collected after centrifugation and protein quantification was determined using BCA method. The samples were resolved by SDS-polyacrylamide gel electrophoresis, transferred to polyvinylfluoride membranes, and incubated with the appropriate primary and secondary antibodies. Finally, the results were detected using the ECL Plus Western blotting system kit (Amersham, Pittsburgh, PA, USA). Primary antibodies were used in our study as follows: mouse anti-Thy-1 (ab225; Abcam, Cambridge, UK), mouse anti-TLR4 (ab22048; Abcam), rabbit anti-total-H3 (ab1791; Abcam), rabbit anti-Ace-H3 (acetyl K9) (ab 10812; Abcam), rabbit anti-total-H4 (ab 10158; Abcam) and rabbit anti-Ace-H4 (acetyl K16) (ab 61240; Abcam). All of the primary antibodies were at a working dilution of 1:1000. Secondary antibodies (1:5000 dilution) were goat antimouse IgG (sc-2005; Santa Cruz Biotechnologies) or goat anti-rabbit IgG (sc-2004; Santa Cruz Biotechnologies, Santa Cruz, CA, USA). The results of immunoblotting were recorded with the Perfection 3490 photo gel imaging systems (Epson, Suwa, Nagano Prefecture, Japan) and analysed with Image Pro PLUS (Media Cybernetics, Bethesda, MD, USA). The expression level of target proteins was normalized to GAPDH.

### ChIP assay

To analyse the histone modification of acetylated H4 (Ace-H4) associated with the promoter region of Thy-1 gene, ChIP assay was performed as previously described [16]. Briefly, the prepared fibroblasts were seeded into a 6-well plate at a density of 2 × 10^6^ cells. The cells were detached, washed with PBS, then cross-linked with formaldehyde and quenched by adding glycine. The prepared cells were lysed to collect DNA, which was sheared by sonication. Afterwards, sheared DNA was incubated with a ChIP-grade rabbit anti-Ace-H4 antibody (acetyl K16) (ab 61240; Abcam). After immunoprecipitation, the cross-linked DNA was released from the antibody–protein–DNA complex, reversed, and then purified with the provided spin column. Quantitative PCR for Thy-1 expression was performed using the Bio-Rad SYBR green PCR kit. The non-immunoprecipitated DNA was used as an input control for ChIP-DNA. The sequences of the Thy-1 primers were as follows: sense, 5′-TCCTCCAAGCCCTGGACTTCATTT-3′, and antisense, 5′-AAGCCCTCATCCTCATTGGATGGT-3′. The real-time PCR primers were as follows: sense, 5′-ACCAAGCCAGATGCCTGAAAGAGA-3′, and antisense, 5′-AAGCCCTCATCCTCATTGGATGGT-3′. The PCR profile was as follows: denaturation at 96°C for 1 min., followed by 40 cycles of 95°C for 30 sec. and annealing at 60°C for 30 sec. The enrichment of the ChIP-DNA was defined as the ratio of the PCR product of ChIP-DNA to the input DNA. All quantitative PCRs were optimized to ensure the PCR products were in the linear range of the amplification.

### Statistical analysis

All data are represented as mean ± SD. SPSS statistical software (IBM, Armonk, NY, USA), version 12.0 was used for mean value comparisons of single-factor multiple samples. The homogeneity of variance data were analysed with the one-factor analysis of variance least squares difference (LSD) test, and the heterogeneity of variance data were analysed with the Kruskal–Wallis rank sum test. Statistical significance was defined as *P* < 0.05.

## Results

### Effect of LPS on lung fibroblast proliferation

**Fig. 1:**
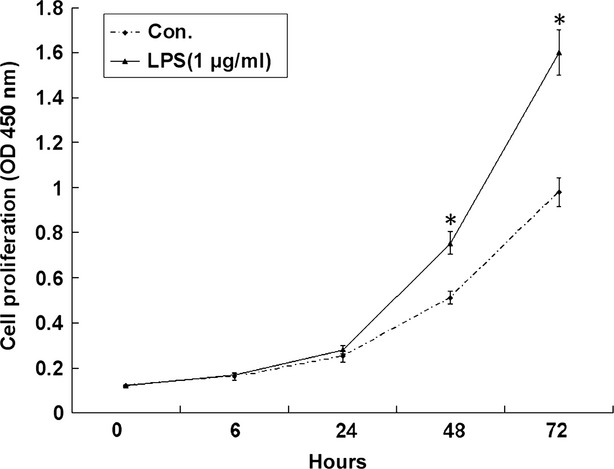
Effect of lipopolysaccharide (LPS) on lung fibroblast proliferation. DNA synthesis in lung fibroblasts at 0, 6, 24, 48 and 72 hrs after LPS challenge was detected by BrdU assay. *P < 0.05 indicates significant difference of the absorbance (OD_450_) between the LPS-challenged cells and the control cells at the same time-point. Points represent mean values (*n* = 3) and error bars represent SD.

To investigate the effect of LPS on lung fibroblastic proliferation during different stages, we measured the DNA synthesis of lung fibroblasts at different time-points from 0 to 72 hrs after LPS challenge using BrdU assay. As shown in Figure 1, at 0, 6 and 24 hrs after LPS challenge, the amount of DNA synthesis was similar to that in the control group at the same time-point, but it significantly increased from 48 to 72 hrs after LPS challenge (*P* < 0.05).

### Dynamic changes of Thy-1 expression in lung fibroblasts in response to LPS challenge

**Fig. 2:**
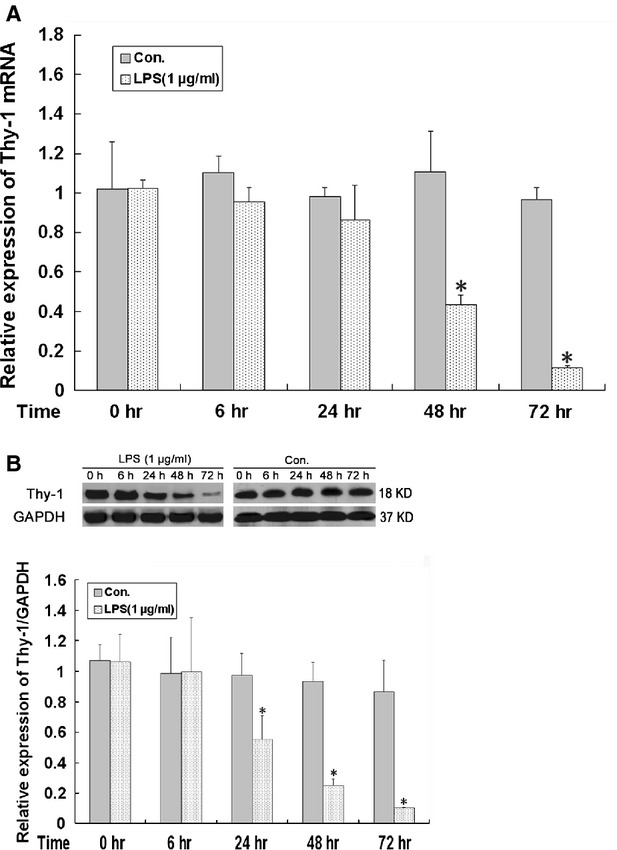
Dynamic changes of Thy-1 expression in lung fibroblast in response to lipopolysaccharide (LPS) challenge. The mRNA of Thy-1 was detected by real-time PCR (**A**) and protein expression of Thy-1 was examined by Western blot (**B**) in lung fibroblasts at 0, 6, 24, 48 and 72 hrs after LPS challenge (1 μg/ml). Columns represent mean values and error bars represent SD. Blots are representative of three independent experiments. *P < 0.05 compared with the control group at the same time-point.

To investigate the changes of Thy-1 expression in lung fibroblast in response to LPS challenge, the expression level of Thy-1 mRNA and protein at different time-point after LPS challenge was examined by real-time PCR and Western blot. The results of Real-time PCR and Western blot confirmed that control lung fibroblasts were predominantly Thy-1-positive. Thy-1 expression in lung fibroblasts decreased from 24 hrs and disappeared between 48 and 72 hrs after LPS challenge, suggesting lung fibroblasts experienced phenotypic transformation from Thy-1(+) cells to Thy-1 (−) one (Fig. 2A and B).

### Effect of TLR4 on Thy-1 expression in lung fibroblasts in response to LPS challenge

**Fig. 3:**
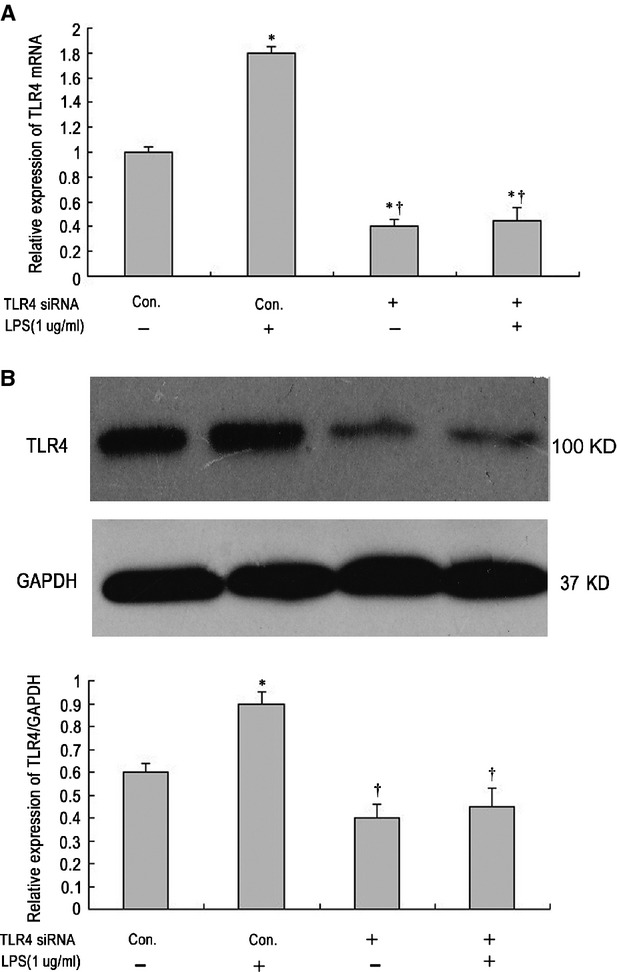
Effect of TLR4 on TLR4 expression in lung fibroblast in response to lipopolysaccharide (LPS) challenge. The effect of siRNA-mediated TLR4 depletion (1 × 10^8^ TU/ml for 48 hrs) on the expression of TLR4 mRNA and protein in lung fibroblasts 72 hrs after LPS challenge by real-time PCR (**A**) and Western blot (**B**) respectively. Columns represent mean values and error bars represent SD. Blots are representative of three independent experiments. *P < 0.05 compared with the negative control group (column 1). †*P* < 0.05 compared with the positive control group (column 2).

To investigate the effect of TLR4 on Thy-1 expression in lung fibroblasts in response to LPS challenge, TLR4 mRNA was depleted with a TLR4-siRNA-lentivirus. Figure 3 showed the effect of siRNA-mediated TLR4 depletion on the expression of TLR4 mRNA (Fig. 3A) and protein (Fig. 3B) in lung fibroblasts 72 hrs after LPS challenge. TLR4 mRNA and protein expression were reduced in lung fibroblasts after TLR4-siRNA-lentivirus infection with an optimized inhibitory dose.

**Fig. 4:**
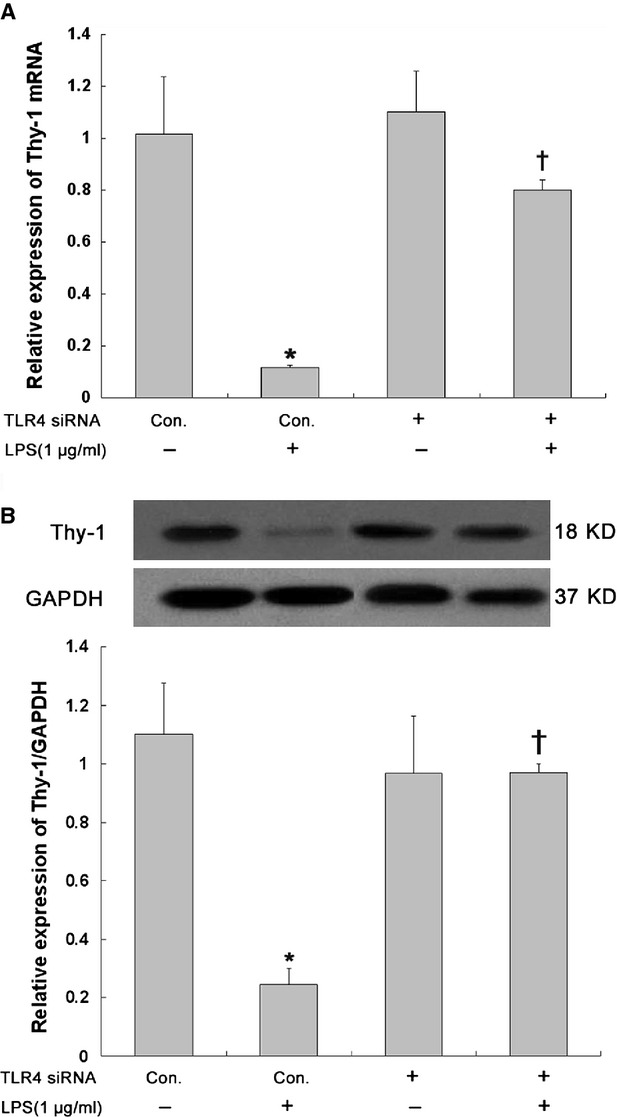
Effect of TLR4 on Thy-1 expression in lung fibroblast in response to lipopolysaccharide (LPS) challenge. The effect of siRNA-mediated TLR4 depletion (1 × 10^8^ TU/ml for 48 hrs) on the expression of Thy-1 mRNA and protein in lung fibroblasts 72 hrs after LPS challenge by real-time PCR (**A**) and Western blot (**B**) respectively. Columns represent mean values and error bars represent SD. Blots are representative of three independent experiments. *P < 0.05 compared with the negative control group (column 1). †*P* < 0.05 compared with the positive control group (column 2).

The expression level of Thy-1 mRNA and protein at 72 hrs after LPS challenge was examined with real-time PCR and Western blot respectively. The expression level of Thy-1 mRNA and protein (Fig. 4A and B) in lung fibroblasts significantly decreased 72 hrs after LPS challenge. However, the LPS-induced Thy-1 expression inhibition was absent upon TLR4 gene knockdown.

### Changes in histone modifications in lung fibroblasts in response to LPS challenge

**Fig. 5:**
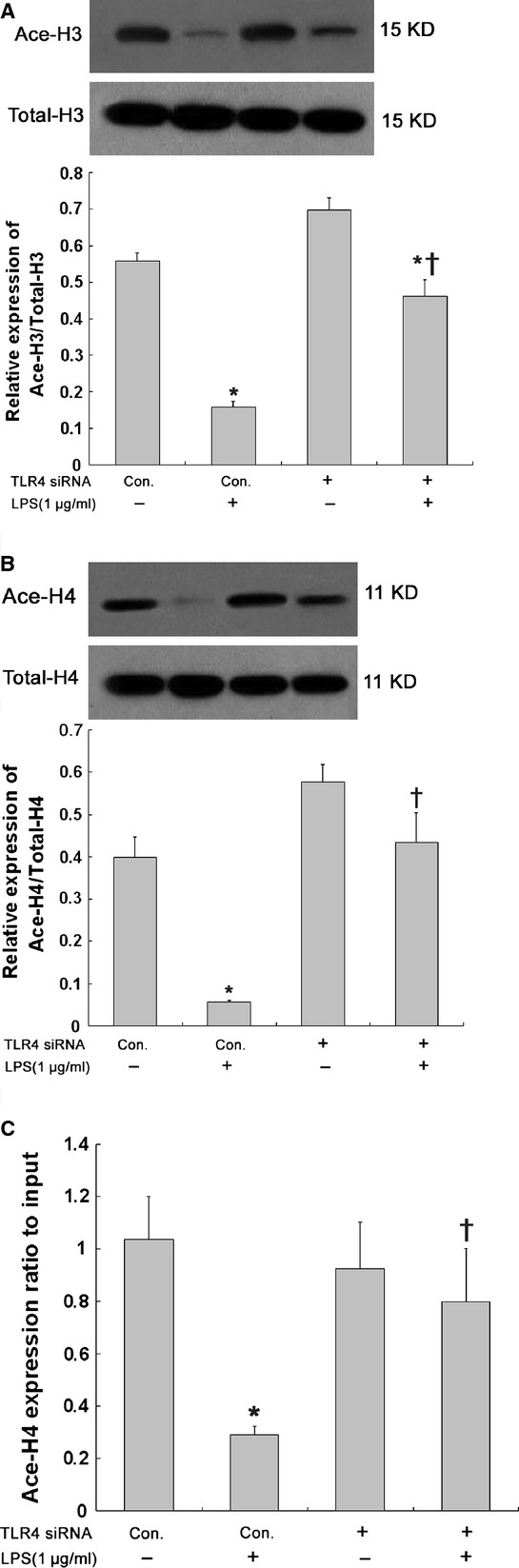
Changes in histone modifications in lung fibroblast in response to lipopolysaccharide (LPS) challenge. Histone deacetylation in lung fibroblasts 72 hrs after LPS challenge was estimated according to the expression of Ace-H3 *versus* Total-H3 (**A**) and Ace-H4 *versus* Total-H4 (**B**) detected by Western blot. Histone modification presented by Ace-H4 (**C**) in the Thy-1 gene promoter region in lung fibroblasts 72 hrs after LPS challenge was detected by the quantitative ChIP assay. The effect of TLR4 on LPS-induced histone deacetylation was evaluated by siRNA-mediated TLR4 depletion (1 × 10^8^ TU/ml for 48 hrs). Columns represent mean values and error bars represent SD. Blots are representative of three independent experiments. *P < 0.05 compared with the negative control group (column 1). †*P* < 0.05 compared with the positive control group (column 2).

Ace-H3 and Ace-H4, as well as Total-H3 and Total-H4 levels were measured, by Western blot, in lung fibroblasts infected with TLR4-siRNA-lentivirus 72 hrs after LPS challenge. Ace-H3 and Ace-H4 decreased the LPS-challenged lung fibroblasts when compared with the non-LPS-challenged cells. Interestingly, compared with non-transfected lung fibroblasts, there was a slight increase in Ace-H3 and Ace-H4 in TLR4-depleted cells after LPS challenge, indicating TLR4 mRNA depletion by RNAi may partially inhibit LPS-mediated reduction of Ace-H3 and Ace-H4 (Fig. 5A and B).

### Changes in histone modification at the Thy-1 promoter region in LPS-challenged lung fibroblasts

To investigate the mechanisms underlying the reduction of Ace-H4 in response to LPS challenge, we examined Ace-H4 in the promoter region of the Thy-1 gene by ChIP assay. Ace-H4 has been reported as a marker for active chromatin structure, enriched Ace-H4 is associated with open chromatin structure and Ace-H4 has been shown in the transcriptional activation of Thy-1. Berger SL found a correlation between Ace-H4 and the Thy-1 promoter region in previous study [23]. As shown in Figure 5C, there was lower amount of precipitated DNA associated with Ace-H4 at the Thy-1 promoter region in the LPS-challenged cells when compared to the control cells or cells transfected with the TLR4-siRNA-lentivirus. These results indicated that Ace-H4 at the Thy-1 promoter region decreased in response to LPS challenge, and suggested that LPS could mediate epigenetic silence of Thy-1 gene.

## Discussion

Although emerging evidences for the association of LPS and lung fibroblast aberrant proliferation during ALI/ARDS have been obtained from *in vivo* and *in vitro* studies, the role of LPS in lung fibroblast proliferation is still uncertain because of unaccountable inconsistent results from similar studies. Therefore, it is necessary to perform further investigation for the related mechanisms. To this end, our study investigated the role of LPS in the epigenetic regulation of Thy-1 expression associated with lung fibroblast proliferation. Our study revealed that LPS could inhibit Thy-1 expression in lung fibroblasts *via* the TLR4 pathway. Furthermore, histone deacetylation in the Thy-1 gene promoter region and sequential transcriptional repression of Thy-1 gene were involved in this process.

In literature, the reports regarding the effect of LPS challenge on fibroblast proliferation are controversial. Some studies showed that LPS could promote cell proliferation in cultured human small intestinal lamina propria fibroblasts [24], adventitial fibroblasts [5, 6], 3T6 fibroblasts [25], human periodontal ligament fibroblasts [26] and lamina propria fibroblasts [27]. However, studies using human gingival fibroblasts and rat embryo fibroblasts yielded opposite results [7–9, 28]. From these reports, we did a comparison of LPS challenge and found that extended duration of LPS challenge was more likely to induce fibroblast proliferation. Zhang *et al*. reported that LPS had a dose-dependent inhibitory effect on fibroblast proliferation 24 hrs after LPS challenge [29]. On the other hand, Yang *et al*. demonstrated that LPS was able to significantly stimulate human skin fibroblast proliferation after more than 6 days of incubation [30]. In this study, we found that LPS challenge, at a concentration of 1 μg/ml, had little impact on the proliferation of cultured mouse lung fibroblasts within 24 hrs, but significantly induced fibroblast proliferation 48–72 hrs after challenge. Our previous study [19] has also verified that LPS could activate lung fibroblasts and induce the fibroblast-myofibroblast transition. As the active form of fibroblast, myofibroblast has the aberrant capability of proliferation, which is closely related to pulmonary fibrosis.

We also observed Thy-1-related lung fibroblast phenotype transformation roughly parallel in time to LPS-induced lung fibroblast proliferation. Different phenotypic heterogeneity is an important factor for controlling propensity of fibroblast to fibrogenic responses. A growing body of research suggests that the absence of Thy-1 in fibroblasts correlates with a more fibrotic phenotype *in vitro* and *in vivo*, such as in the bleomycin-induced pulmonary fibrosis model and human IPF [11, 31, 32]. Therefore, it is reasonable to infer that LPS can promote lung fibroblast proliferation through Thy-1-related phenotype transformation. This may partly explain why LPS can induce different proliferative reactions during different periods after fibrogenic challenge in the same tissue.

Fewer studies have been reported on the mechanisms related to gene expression and regulation of Thy-1, a glycophosphatidylinositol (GPI)-linked cell-surface glycoprotein expressed on fibroblasts. It has been reported that the loss of fibroblast Thy-1 surface expression, induced by fibrogenic cytokines interleukin-1 and tumour necrosis factor-alpha, is associated with Thy-1 shedding [11]. In 2008, Sanders *et al*. revealed that promoter region hypermethylation resulted in the silence of Thy-1 expression in fibroblastic foci in patients with IPF, suggesting an involvement of epigenetic regulation in fibrotic phenotype programming [12]. In 2011, their further study demonstrated the epigenetic silencing of Thy-1 by histone deacetylation at the Thy-1 gene promoter region in lung fibroblasts [16]. Acetylation of lysine residues at N-terminal domains of core histones is associated with Thy-1 gene transcriptional activation in lung fibroblasts. In contrast, histone deacetylation is associated with Thy-1 gene transcriptional silencing [16]. Aung *et al*. revealed that LPS regulated pro-inflammatory gene expression in macrophages by altering histone deacetylase expression [17]. Angrisano *et al*. also reported that LPS could induce IL-8 activation in human intestinal epithelial cells through histone modification including H3 acetylation and H3K4, H3K9 and H3K27 methylation [18]. In this study, we demonstrated the deacetylation of H3 and H4 and inhibition of Thy-1 gene transcription after LPS challenge in cultured mouse lung fibroblasts, indicating that LPS may induce Thy-1 gene transcriptional silencing through histone deacetylation at the Thy-1 gene promoter region in lung fibroblasts. This would be an essential mechanism for LPS-induced and Thy-1-related phenotype transformation in lung fibroblasts. Moreover, histone modification includes, but is not limited to acetylation. Histone methylation may also play an important role during the process of Thy-1 gene transcriptional silencing, which will be further investigated in our future studies.

Our previous studies found that LPS-induced lung fibroblast proliferation was associated with its receptor, TLR4. In this study, we also found that histone deacetylation at the Thy-1 gene promoter region in lung fibroblasts was reduced to some extent in the TLR4-depleted cells, suggesting that histone deacetylation at the Thy-1 gene promoter region is involved in the TLR4-mediated transcriptional inhibition of the Thy-1 gene, but the intrinsic mechanisms are still not clear. Histone deacetylation is mainly regulated by histone deacetylase (HDAC), a class of enzymes that control gene expression by removing acetyl groups from lysine residues in histone molecules. The excessive expression and activation of HDAC closely correlates with Thy-1 gene silencing. Sanders *et al*. reported that histone deacetylase inhibitor trichostatin A (TSA) restored Thy-1 expression in Thy-1 (−) lung fibroblasts, and this process was associated with enrichment of histone acetylation [16]. Recent studies found that LPS could induce a number of HDACs (Hdac-4, 5, 7) in murine bone marrow-derived macrophages (BMM) [17]. In addition, LPS also increased HDAC activity and stimulated TNF-alpha expression *via* the accumulation of NF-kappa B/p65 at the TNF-alpha promoter in cardiomyocytes [33]. It was reported that histone deacetylase inhibitors could attenuate LPS-induced acute lung injury [34] or growth arrest human rheumatoid arthritis synovial fibroblastic E11 cells through suppressing LPS-induced NF-kappa B p65 nuclear accumulation [35]. Furthermore, the relationship between TLR4 signalling activation and histone deacetylation was shown in several reports [36, 37]. HDAC inhibitors suppressed the expression of numerous host defence genes, which were stimulated by TLR agonists [36], and the expression of innate antiviral molecules, such as IFN beta, interferon-simulated genes and proteins involved in TLR3/TLR4 signalling [38]. HDAC inhibitors have also been shown to inhibit TLR4-dependent activation and function of macrophages and dendritic cells (DCs) [37]. According to our findings in this study, we hypothesize that LPS-induced TLR4 signalling activation inhibits lung fibroblast proliferation by regulating HDAC expression or activation. However, further investigation is needed.

Our studies showed that epigenetic regulation of Thy-1 gene expression were involved in LPS-induced lung fibroblast proliferation through histone modification in the Thy-1 gene promoter region and sequential lung fibroblast phenotype transformation. Our results suggest that Thy-1-related lung fibroblast phenotype transformation contributes to the pathogenesis of LPS-induced lung fibroblast proliferation and pulmonary fibrosis.
